# Deep anterior lamellar keratoplasty versus penetrating keratoplasty: comparison of clinical outcomes in contralateral eyes

**DOI:** 10.5935/0004-2749.20230053

**Published:** 2023

**Authors:** Nesrin Tutaş Günaydın, Berkay Akmaz, Baran Kandemir

**Affiliations:** 1 Department of Ophthalmology, Kartal City Hospital, University of Health Sciences Dr. Lutfi Kırdar, Istanbul, Turkey.; 2 Department of Ophthalmology, Manisa City Hospital, Manisa, Turkey.

**Keywords:** Corneal diseases/surgery, Keratoconus/surgery, Keratoplasty, penetrating/methods, Corneal transplantation/methods, Intraocular pressure, Comparative study, Doenças da córnea/cirurgia, Ceratocone/cirurgia, Ceratoplastia penetrante/métodos, Transplante de córnea/métodos, Pressão intraocular, Estudo comparativo

## Abstract

**Purpose:**

This study aimed to compare the clinical outcomes following deep anterior lamellar keratoplasty and penetrating keratoplasty in contralateral eyes of the same patients.

**Methods:**

In this retrospective, comparative case series, clinical outcome data included best-corrected visual acuity, refractive spherical equivalent, refractive astigmatism, endothelial cell density, endothelial cell loss, central corneal thickness, and intraocular pressure, which were evaluated at 6, 12, 24, and 36 months after deep anterior lamellar keratoplasty and penetrating keratoplasty. Additionally, complications were assessed.

**Results:**

Fifty-two eyes (26 patients) were included, of which 19 patients had keratoconus, 6 had stromal dystrophy, and 1 had post-laser-assisted *in situ* keratomileusis ectasia. The mean follow-up was 44.1 ± 10.5 months in the deep anterior lamellar keratoplasty Group and 47.9 ± 11.9 months in the penetrating keratoplasty Group. No significant differences were observed in the mean best-corrected visual acuity, refractive spherical equivalent, refractive astigmatism, and central corneal thickness between the deep anterior lamellar keratoplasty and penetrating keratoplasty Groups during follow-up. The endothelial cell density was significantly higher in the deep anterior lamellar keratoplasty Group than in the penetrating keratoplasty Group at 24 and 36 months postoperatively (p=0.022 and 0.013, respectively). Endothelial cell loss was significantly lower in the deep anterior lamellar keratoplasty Group than in the penetrating keratoplasty Group at 24 and 36 months postoperatively (p=0.025 and 0.001, respectively). Intraocular pressure was significantly lower in the deep anterior lamellar keratoplasty Group than in the penetrating keratoplasty Grroup at 6 months postoperatively (p=0.015). Microperforation occurred in 4 eyes (15%) during deep anterior lamellar keratoplasty surgery; however, penetrating keratoplasty was not required. No endothelial rejection occurred in the penetrating keratoplasty Group during follow-up.

**Conclusions:**

Over the 3-year follow-up, endothelial cell loss and intraocular pressure in the deep anterior lamellar keratoplasty Group were significantly lower than those in the penetrating keratoplasty Group, while visual and refractive results were similar.

## Abstract (pt)

**Objetivo:**

Este estudo teve como objetivo comparar os resultados clínicos após ceratoplastia lamelar anterior profunda e ceratoplastia penetrante nos olhos contralaterais dos mesmos pacientes.

**Métodos:**

Nesta série de casos comparativa e retrospectiva, avaliaram-se os seguintes dados de resultados clínicos: melhor acuidade visual corrigida, equivalente esférico refrativo, astigmatismo refrativo, densidade de células endoteliais, perda de células endoteliais, espessura central da córnea e pressão intraocular. Esses dados foram avaliados aos 6, 12, 24 e 36 meses após ceratoplastia lamelar anterior profunda e ceratoplastia penetrante. Também foram avaliadas as complicações.

**Resultados:**

Foram incluídos 52 olhos (26 pacientes), sendo que 19 pacientes apresentavam ceratocone, 6 apresentavam distrofia estromal e 1 apresentava ectasia após ceratomileuse *in situ assistida por laser.* O tempo médio de acompanhamento foi de 44,1 ± 10,5 meses no grupo da ceratoplastia lamelar anterior profunda e 47,9 ± 11,9 meses no grupo da ceratoplastia penetrante. Nenhuma diferença significativa foi observada nas médias da melhor acuidade visual corrigida, equivalente esférico refrativo, astigmatismo refrativo e espessura central da córnea entre os grupos da ceratoplastia lamelar anterior profunda e da ceratoplastia penetrante durante o acompanhamento. A densidade de células endoteliais foi significativamente maior no grupo da ceratoplastia lamelar anterior profunda que no grupo da ceratoplastia penetrante aos 24 e 36 meses de pós-operatório (p=0,022 e 0,013, respectivamente). A perda de células endoteliais foi significativamente menor no grupo da ceratoplastia lamelar anterior profunda que no grupo da ceratoplastia penetrante aos 24 e 36 meses de pós-operatório (p=0,025 e 0,001, respectivamente). A pressão intraocular foi significativamente menor no grupo da ceratoplastia lamelar anterior profunda que no grupo da ceratoplastia penetrante aos 6 meses de pós-operatório (p=0,015). Ocorreu microperfuração em 4 olhos (15%) durante a cirurgia de ceratoplastia lamelar anterior profunda; entretanto, a ceratoplastia penetrante não foi necessária. Não ocorreu nenhuma rejeição endotelial no grupo da ceratoplastia penetrante durante o período de acompanhamento.

**Conclusões:**

Durante o acompanhamento de 3 anos, a perda de células endoteliais e a pressão intraocular foram significativamente menores no grupo da ceratoplastia lamelar anterior profunda que no grupo da ceratoplastia penetrante, mas os resultados visuais e refrativos foram semelhantes.

## INTRODUCTION

Penetrating keratoplasty (PK) has been the main surgical treatment option for corneal stromal and endothelial diseases^(1,2)^. Long-term follow-up studies on PK have reported good visual results, usually after 18-24 months^(1,3)^. However, immunological rejection develops in 18%-34% of patients with PK, leading to endothelial cell loss (ECL) and subsequent graft failure^(4,5)^.

Deep anterior lamellar keratoplasty (DALK) is an alternative surgical technique to PK to treat various corneal stromal diseases with healthy endothelium^(6)^. The main advantages of DALK, compared to PK, include reduction in intraocular complications, such as iris prolapse, choroidal effusion, suprachoroidal hemorrhage, endophthalmitis, and the risk of endothelial graft rejection^(7-9)^. In DALK surgery, the globe is more resistant to blunt trauma since the Descemet’s membrane and corneal endothelium remain in place; furthermore, steroids could be used for a shorter period, and earlier suturing can be performed, as the wound heals earlier^(10)^. However, the challenges with DALK include the time-consuming and technically demanding nature of the procedure.

Owing to these procedures varying advantages and challenges, their clinical outcomes often differ. Consequently, these procedures have been compared between different patient groups in the literature. However, comparison with a contralateral eye in the same patient is a better approach for evaluating objective outcomes between surgical techniques than is a comparison across patients. Although a few previous studies have performed such comparisons using contralateral eyes, their sample sizes were limited^(6,11,12)^.

Therefore, this study aimed to report the clinical outcomes of a larger cohort of patients who underwent DALK in one eye and PK in the contralateral eye.

## METHODS

The medical records of 26 patients (52 eyes) who underwent DALK in one eye and PK in the contralateral eye were reviewed retrospectively at the University of Health Science Kartal City Hospital from 2010 to 2017. In contralateral eyes, PK was performed in keratoconus diseases with Descemet’s membrane rupture (hydrops history and corneal opacities). Moreover, PK was planned in patients with corneal dystrophy with an advanced and deep lesion on the anterior segment optical coherence tomography.

This study included eyes with a follow-up period of at least 3 years after the surgery, normal intraocular pressure (IOP) before surgery, and no antiglaucoma medication. The study did not include eyes with ocular comorbidities and those requiring ocular surgery, such as cataract surgery, and keratoplasty. All surgeries were performed by the same surgeon (BK). The University of Health Sciences provided all donor corneal buttons, Dr. Lütfi Kırdar Kartal City Hospital Eye Bank, and were stored in the short-term storage solution Eusol-C^®^ (Corneal Chamber, Alchimia, Ponte San Nicolo, Italy) at 4°C. The total storage times of the donor corneas were noted separately for both PK and DALK surgery. Endothelial cell density (ECD) of the donor cornea was measured using a specular microscope (Konan Eye Bank KeratoAnalyzer EKA-04, Hyogo, Japan) provided by the University of Health Sciences, Dr. Lütfi Kırdar Kartal City Hospital Eye Bank, Istanbul.

The data collected included preoperative and postoperative best spectacle-corrected visual acuity (BCVA), refractive spherical equivalent (RSE), refractive astigmatism (RA), ECD, ECL, central corneal thickness (CCT), IOP, and complications. Visual acuity was measured using the standard Snellen chart, and results were converted into logarithm of the minimum angle of resolution (logMAR) units for statistical analysis. Postoperative ECD was measured using a noncontact specular microscope (Topcon SP-2000P; Topcon Corporation, Tokyo, Japan). CCT was measured by ultrasonic pachymetry (Tomey SP-3000; Tomey GmbH, Erlangen, Germany), and IOP was measured with Goldmann applanation tonometer (Haay-Streit AG, Koeniz, Switzerland) or Tono-Pen^®^ XL (TPXL; Reichert Technologies, Depew, NY, USA).

This study was conducted in accordance with the Declaration of Helsinki, and approval was obtained from the Institutional Review Board of the University of Health Sciences, Dr. Lutfi Kırdar Kartal City Hospital (protocol number, 8951337). All patients were informed about the advantages and disadvantages of the procedures. Written informed consent was obtained from all patients before the surgeries.

### Surgical techniques

Penetrating keratoplasty

In the PK group, the cornea was trephined using Barron’s vacuum trephine (Katena Products, Denville, NJ, USA) (range, 7.00-8.25 mm), and the donor cornea was punched using Barron’s punch trephine (Katena Products) at a size that was 0.25 µm larger than the recipient cornea (range, 7.25-8.50 mm). The donor cornea was sutured to the recipient bed using 10-0 nylon sutures with 16 interrupted or a combination of 8 interrupted and 16 continuous sutures.

Deep anterior lamellar keratoplasty

As described previously, DALK was based on Anwar and Teichmann’s big bubble technique^(13)^. The cornea was trephined at partial thickness with on’s vacuum trephine to 70%80% of its depth (range, 7.00-8.25 mm). Air was injected into the deep stroma by a 30-gauge needle to create a large bubble. The anterior stromal tissue was removed with a crescent knife. Following the air collapse with a 45-degree knife, the remaining stromal tissue was stripped up to Descemet’s membrane by dividing it into four quadrants. The endothelium of the donor corneal buttons was made visible by 0.06% trypan blue (Ocublu-Try, Bursa, Turkey), and it was removed partially from the stromal bed using forceps. Barron’s punch trephine punched donor tissue at a size that was 0.25 µm larger than the recipient cornea (range, 7.25-8.50 mm), and the donor cornea was sutured in the same manner as that for the PK technique.

### Postoperative management

Standardized eye examinations were conducted preoperatively and at 1 day, 1 week, and 1, 3, 6, 12, 24, and 36 months postoperatively and thereafter, following the standard care protocol of our clinic for keratoplasty patients. All eyes were treated with 0.5% moxifloxacin hydrochloride (Vigamox^®^; Alcon Pharma GmbH, Freiburg, Germany) and 0.1% dexamethasone (Maxidex®; Alcon Pharma GmbH) five times daily after surgery. The topical antibiotic was discontinued after 10 days. Dexamethasone was replaced with 0.5% loteprednol etabonate (Lotemax^®^; Bausch & Lomb, Bridgewater, NJ, USA) four times daily by 3-6 months after PK and 3 months after DALK. Topical steroid therapy was continuously administered for at least 18 months and a maximum of 2 years following PK, and for at least 12 months and a maximum of 18 months following DALK, which was gradually reduced to at least once a day according to patient’s clinical outcomes.

The postoperative corneal astigmatism was followed up with topography (Sirius Scheimpflug-Placido topographer; Costruzione Strumenti Oftalmici, Florence, Italy). To reduce postoperative RA values and improve BCVA, selective suture removal was performed based on topographic maps. Antibiotic eye drops were used for 10 days after suture removal.

### Statistical analysis

Statistical analyses were performed using SPSS software (SPSS Version 21.0.; IBM Corp., Armonk, NY, USA). The Kolmogorov-Smirnov test was used to check the normality of data distribution. For parametric analysis, Student’s *t*-test was used to evaluate differences between the DALK and PK groups. When parametric analysis could not be performed, the differences between the PK and DALK groups were evaluated by the Mann-Whitney *U* test. P-value <0.05 was considered statistically significant.

## RESULTS

The present study included 52 eyes of 26 patients (8 men and 18 women) with an average age of 31.3 ± 8.9 (range, 12-50) years. Nineteen patients had keratoconus, 6 had stromal dystrophy, and 1 had post-laser-assisted *in situ* keratomileusis ectasia. The mean total preservation time for the donor corneas was 4.2 ± 2.3 days (range, 1-10 days) for PK and 4.5 ± 2 days (range, 2-9 days) for DALK surgery. There were no significant differences in the preservation times for the donor corneas between the surgery groups.

The mean follow-up time was 44.1 ± 10.5 (range, 38-98) months for the DALK group and 47.9 ± 11.9 (range, 41-131) months for the PK group (p=0.23). The mean time elapsed between surgery and complete suture removal was 11.38 ± 2.82 (range, 8-20) months and 16.55 ± 5.91 (range, 12-28) months in the DALK and PK groups, respectively.

### Visual outcomes

**Figure 1:**
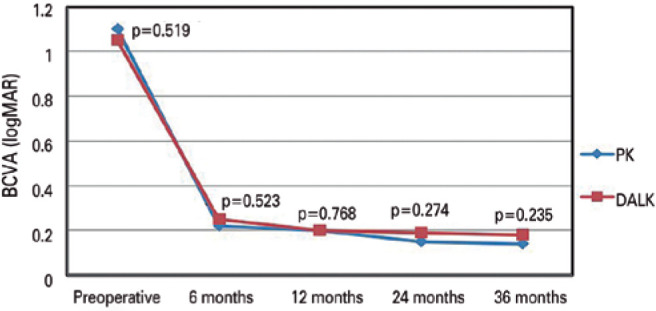
Changes in BCVA (logMAR) values after DALK and PK with postoperative 3-year BCVA, as examined with independent sample *t-*tests.

The preoperative logMAR BCVA was 1.05 ± 0.27 in the DALK group and 1.1 ± 0.27 in the PK group. At 6 months postoperatively, the logMAR BCVA had improved to 0.25 ± 0.20 in the DALK group and 0.22 ± 0.14 in the PK group. Thereafter, the logMAR BCVA remained virtually unchanged, as follows: at 12 months, 0.20 ± 0.17 in the DALK group and 0.20 ± 0.10 in the PK group; at 24 months, 0.19 ± 0.16 in the DALK group and 0.15 ± 0.11 in the PK group; and at 36 months, 0.18 ± 0.12 in the DALK group and 0.14 ± 0.13 in the PK group. No significant difference was noted in BCVA between the DALK and PK groups during the follow-up (Figure 1).

BCVA= best-corrected visual acuity; logMAR= logarithm of the minimum angle of resolution; DALK= deep anterior lamellar keratoplasty; PK= penetrating keratoplasty.

### Refractive outcomes

There were no significant differences in the RSE and RA values between the DALK and PK groups at any follow-up visits (independent sample *t-*tests; Table 1).

**Table 1:** Comparison of refractive outcomes between the groups

Group	6 months		12 months		24 months		36 months	
	RSE (D)	RA (D)	RSE (D)	RA (D)	RSE (D)	RA (D)	RSE (D)	RA (D)
PK	-5.63 ± 3.95	-4.15 ± 3.44	-5.27 ± 3.46	-4.02 ± 3.14	-5.02 ± 3.05	-3.75 ± 2.75	-4.62 ± 2.78	-3.50 ± 2.70
DALK	-5.86 ± 3.28	-3.79 ± 3.40	-5.70 ± 3.16	-3.79 ± 3.40	-5.53 ± 2.45	-3.45 ± 3.21	-4.76 ± 1.97	-3.36 ± 2.40
p-value	0.820	0.801	0.641	0.801	0.752	0.788	0.834	0.844

Data are presented as the mean ± standard deviation and were analyzed by independent sample *t-*tests.

PK= penetrating keratoplasty; DALK= deep anterior lamellar keratoplasty; RSE= refractive spherical equivalent; RA= refractive astigmatism.

IOP= intraocular pressure; DALK= deep anterior lamellar keratoplasty; PK= penetrating keratoplasty.

### Endothelial cell density

**Figure 2:**
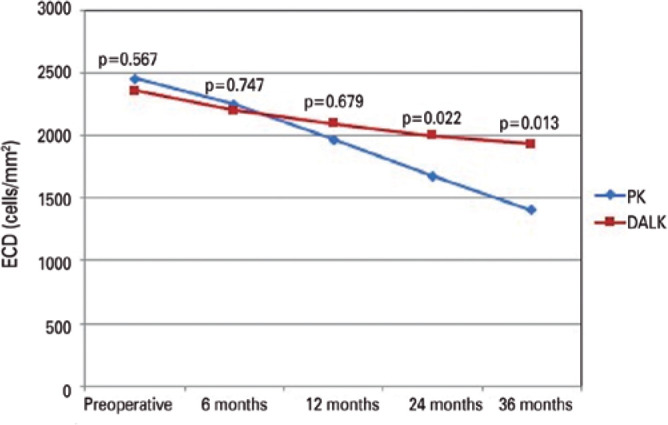
Changes in the ECD in the DALK and PK groups with postoperative 3-year ECD, as assessed using independent sample *t-*tests.

Table 2 and figure 2 show the ECD outcomes of both surgeries. No significant differences were observed between the groups in the mean ECD preoperatively and at 6 and 12 months postoperatively. The ECD was significantly higher in the DALK group than in the PK group at 24 and 36 months postoperatively. At 24 and 36 months postoperatively, the ECL was significantly lower in the DALK group than in the PK group (Mann-Whitney *U* tests; Table 3).

**Table 2:** Comparison of preoperative and postoperative endothelial cell density values between the groups

Group	Preoperative	6 months	12 months	24 months	36 months
PK	2453.9 ± 573.4	2246.8 ± 583.4	1965.3 ± 683.4	1676.2 ± 388.8	1406.2 ± 352.7
DALK	2356.7 ± 642.6	2198.6 ± 486.8	2093.5 ± 612.2	1999.5 ± 287.4	1931.5 ± 242.2
p-value	0.567	0.747	0.679	0.022	0.013

Data are presented as the mean ± standard deviation (cells/mm^2^) and were analyzed by independent sample *t*-tests.Values in bold are statistically significant.

PK= penetrating keratoplasty; DALK= deep anterior lamellar keratoplasty.

ECD= endothelial cell density; DALK= deep anterior lamellar keratoplasty; PK= penetrating keratoplasty.

**Table 3:** Comparison of endothelial cell loss outcomes between the groups

Group	6 months	12 months	24 months	36 months
PK	8,43 ± 4.56	19,91 ± 10,2	24,91 ± 12,2	31,69 ± 14.6
DALK	6,70 ± 3.21	11,16 ± 5,8	15,15 ± 7.89	18,00 ± 8.58
p*-value*	0.431	0.174	**0.025**	**0.001**

Data are presented as the mean ± standard deviation (%) and were analyzed by Mann-Whitney *U* tests. Values in bold are statistically significant.

PK= penetrating keratoplasty; DALK= deep anterior lamellar keratoplasty.

### Central corneal thickness and intraocular pressure

During the follow-up, CCT was not significantly different between the surgical groups (independent sample *t-*tests). Table 4 shows the mean CCT values.

**Table 4:** Comparison of central corneal thickness between the groups

Group	Preoperative	6 months	12 months	24 months	36 months
PK	461.36 ± 36.49	548.21 ± 38.43	551.65 ± 34.76	549.78 ± 39.21	544.34 ± 41.23
DALK	453.41 ± 38.91	562.12 ± 41.67	558.38 ± 40.55	551.87 ± 41.43	546.30 ± 37.05
p-value	0.621	0.599	0.435	0.278	0.321

Data are presented as the mean ± standard deviation (µ) and were analyzed by independent sample *t*-tests.

PK= penetrating keratoplasty; DALK= deep anterior lamellar keratoplasty.

**Figure 3:**
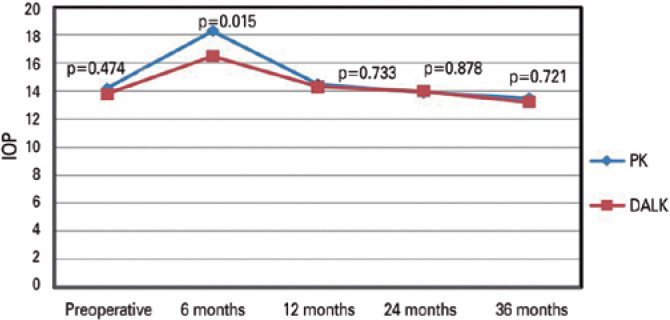
Changes in IOP in the DALK and PK groups with postoperative 3-year IOP, as assessed with independent sample *t-*tests.

The preoperative IOP did not exceed 21mmHg in any patient. At 6 months following the surgery, the IOP was significantly lower in the DALK group than in the PK group (independent sample *t-*test; Figure 3).

### Complications

No intraoperative complications were observed in the PK group. Microperforation of the Descemet’s membrane occurred in 4 eyes (15%) during the DALK surgery; however, this did not require conversion to PK. In 3 eyes (11%), a double anterior chamber was observed and resolved after intracameral air injection at 1 day postoperatively.

Thirty-two months after surgery, IOP elevation was recorded in 1 eye (3.84%, n=26) in the PK group. The IOP increased to 30 mmHg and responded well to topical antiglaucoma therapy.

Adjustment of sutures was performed in 7 eyes (13%): at 3 months postoperatively in 5 eyes (19%) in the DALK group, and at 5 and 6 months postoperatively in 2 (7%) eyes in the PK group. Graft failure and rejection did not occur during the follow-up in any of the cases in the study groups. There were no cases of retransplantation.

## DISCUSSION

This study directly compared the results of DALK and PK in the contralateral eyes of the same patients in a sizeable patient cohort. We observed no significant differences in the mean BCVA, RSE, RA, and CCT between the DALK and PK groups during the follow-up. The ECD was significantly higher, and ECL was significantly lower in the DALK group than in the PK group at the postoperative 24- and 36-month follow-up visits. Moreover, the IOP was significantly lower in the DALK group than in the PK group at 6 months postoperatively.

The comparative visual and refractive outcomes following DALK and PK have been reported by several studies^(9,14-17)^. Our finding of no differences in the visual and refractive outcomes between these two techniques was consistent with the results of several other studies that performed the comparisons across different patients^(12,14,18)^. Moreover, similar results have been reported for BCVA, RSE, and RA in the contralateral eyes of the same patients in previous studies on DALK and PK^(6,11,12)^. In contrast, other prior studies have shown better visual and refractive outcomes in PKP-operated groups than in DALK-operated groups^(2,12,15)^. This discrepancy in findings could be due to the irregularity of the host-donor interface or the use of different techniques that lead to high residual bed thickness. All DALK procedures were successfully completed with Anwar and Teichmann’s big bubble technique in our series. With this technique, Descemet’s membrane is revealed more clearly, providing a smoother surface between the recipient’s and donor’s cornea^(13)^. The visual and refractive results were statistically similar in both groups at the final follow-up, possibly owing to the complete stromal removal in the DALK eyes.

ECD is among the most important factors ensuring the long-term survival of the graft in keratoplasty^(8)^. However, previous studies have shown that PK results in accelerated ECL^(2,19,20)^. A mean relative annual loss of endothelial cells of between 12% and 17% has been reported^(21)^. Borderie et al.^(22)^ reported ECLs of 53% and 61% at 5 and 10 years postoperatively, respectively, in their PK group. In a corneal donor study, the median ECL with clear grafts at 5 years postoperatively was 70%^(23)^. ECD gradually decreases over an average of 20 years, and ECL is more pronounced in the first years after PK^(24)^. This suggests that the cornea has an average lifespan of 20 years after PK. This may be particularly important in individuals with a longer life expectancy, such as patients with keratoconus. Unlike in PK, the recipients’ Descemet’s membrane and endothelium are maintained in DALK. Therefore, it is considered that the endothelium is less damaged in a standard DALK surgery than in PK surgery^(13)^. Bahar et al.^(19)^ reported ECL rates of 43% in a PK group and 6% in a DALK group following 1 year. Fontana et al.^(25)^, who performed DALK in keratoconus eyes using the big bubble technique, reported a postoperative ECL rate of 6% per year. Our study demonstrated no significant difference in the ECD between the DALK and PK groups at 1, 6, and 12 months postoperatively, although there was a significant difference in the ECD between the two groups at 24 and 36 months postoperatively, whereby the ECD was higher in the DALK group than in the PK group. Further, in a study with a design similar to ours (contralateral eyes in 8 patients), the authors reported that ECD values were significantly higher in the DALK group than in the PK group at 12 and 24 months postoperatively^(6)^. Shimazaki et al.^(26)^, in randomized controlled trials comparing DALK and PK in different patients, revealed that the corneal ECD in DALK-operated eyes stabilized after 6 months. In a randomized multicenter clinical trial, Cheng et al.^(27)^ showed continued ECL during follow-up in DALK and PK groups, with the ECL being significantly higher in the PK group than in the DALK group at 12 months postoperatively. In the current study, when comparing the DALK and PK groups, the ECL was lower in the DALK group (6.70%) than in the PK group (8.43%) at 6 months postoperatively. These figures were 11.16% versus 19.91% at 12 months, 15.15% versus 24.91% at 24 months, and 18% versus 31.69% at 36 months. ECL appears to be the most pronounced in the early postoperative period (6 and 12^th^ months) and then occurs slowly over time. Kubaloğlu et al.^(16)^ reported that the rate of ECL in a DALK group was 8.1% over 1 year, 10.5% over 2 years, and 15.1% over 6 years, which were similar to our DALK results.

In our study, the mean IOP was significantly higher in the PK group than in the DALK group at 6 months postoperatively. This finding was similar to that of a previous study, which reported that the average period between surgery and the first IOP elevation was 5 months after PK^(28)^. The relationship between the use of topical steroids and IOP elevation after keratoplasty is known^(29)^. In the present study, steroid potency was lower, and the duration of steroid use was shorter in the DALK group than in the PK group. This may explain why, at 6 months postoperatively, the IOP was significantly lower in the DALK group than in the PK group. In addition, we found that the rate of IOP elevation in the PK group was 3.84%. However, the incidence of ocular hypertension after PK in the range of 11%-50% has been reported^(29,30)^. The most likely reason for the lower rate of IOP elevation in our study was that our series consisted of patients with non-inflammatory diseases, such as keratoconus, corneal dystrophy, and corneal ectasia. Moreover, the fact that our series comprised these low-risk diseases for rejection could be established by the absence of graft rejection in any of our patients. The small sample size of our series could also have reduced the likelihood of graft rejection. Probably, we did not see graft rejection in the PK group because of the long-term steroid therapy that we routinely use in our patients. In contrast, one of the most important advantages of the DALK surgery performed in half of our series is the absence of endothelial graft rejection^(16)^.

As an early complication, loose sutures were observed more frequently in the DALK group than in the PK group. Rapid healing of the graft-host junction in the DALK group can facilitate the loosening of the sutures. Furthermore, the sutures may loosen owing to the surgeon’s superficial placement of the suture to avoid Descemet’s membrane perforation^(19)^.

A limitation of this study was its retrospective design. Another limitation of the study was the heterogeneity of the diagnoses included in the analysis (keratoconus, stromal corneal dystrophy, post-laser assisted *in situ* keratomileusis ectasia, etc.). However, these heterogeneous diagnoses have been studied due to the small sample size. Therefore, the results should be interpreted after considering this heterogeneity.

In conclusion, our study demonstrated that DALK surgery is a more advantageous procedure than PK in terms of long-term ECL, although it provides similar visual (BCVA) and refractive results. This may be particularly important in individuals with a long life expectancy.
